# Trans-translation is essential in the human pathogen *Legionella pneumophila*

**DOI:** 10.1038/srep37935

**Published:** 2016-11-28

**Authors:** Romain Brunel, Xavier Charpentier

**Affiliations:** 1 Univ Lyon, Université Claude Bernard Lyon 1, INSA-Lyon, CNRS, UMR5240, Microbiologie, Adaptation et Pathogénie, 10 rue Raphaël Dubois, F-69622, Villeurbanne, France; 2 CIRI, Team “Horizontal gene transfer in bacterial pathogens”, Inserm, U1111, Université Claude Bernard Lyon 1, CNRS, UMR5308, École Normale Supérieure de Lyon, Univ Lyon, 69100, Villeurbanne, France

## Abstract

Trans-translation is a ubiquitous bacterial mechanism for ribosome rescue in the event of translation stalling. Although trans-translation is not essential in several bacterial species, it has been found essential for viability or virulence in a wide range of pathogens. We describe here that trans-translation is essential in the human pathogen *Legionella pneumophila*, the etiologic agent of Legionnaire’s disease (LD), a severe form of nosocomial and community-acquired pneumonia. The *ssrA* gene coding for tmRNA, the key component of trans-translation, could not be deleted in *L. pneumophila*. To circumvent this and analyse the consequences of impaired trans-translation, we placed *ssrA* under the control of a chemical inducer. Phenotypes associated with the inhibition of *ssrA* expression include growth arrest in rich medium, hampered cell division, and hindered ability to infect eukaryotic cells (amoebae and human macrophages). LD is often associated with failure of antibiotic treatment and death (>10% of clinical cases). Decreasing tmRNA levels led to significantly higher sensitivity to ribosome-targeting antibiotics, including to erythromycin. We also detected a higher sensitivity to the transcription inhibitor rifampicin. Both antibiotics are recommended treatments for LD. Thus, interfering with trans-translation may not only halt the infection, but could also potentiate the recommended therapeutic treatments of LD.

Translation of new proteins from messenger RNAs (mRNAs) is essential to maintain homeostasis of the cell and the ribosome is a proven target for antibiotics, as full and generalised arrest of translation generally leads to bacteriostasis and ultimately death^1^,^2^. Transient translation arrest occurs naturally but without consequences on the cell viability. It has been shown that ribosome pausing for a few seconds occurs naturally during translation^3^ and that total ribosome blocking (called “stalling”) occurs naturally at a very high rate in bacteria^4^. Common causes of ribosome stalling include translation of specific peptides that interact with the ribosome exit tunnel^5^, stretches of repeated or rare codons that exhaust the local pool of corresponding tRNAs^6^,^7^, and amino acid starvation that reduces the global pool of available charged tRNAs^8^. Besides, a frequent cause of ribosome stalling is the translation of mRNAs lacking a stop codon, as recognition of the stop signal by termination factors is necessary for the release of the translation complex^9^. Such non-stop mRNAs may arise by abortion of transcription and the absence of a quality check between the coupled transcription and translation steps. Non-stop mRNAs are also generated during ribosome pausing by mRNA cleavage near the ribosomal A site^10^ or by ribonucleolytic toxins^11^,^12^,^13^. While most causes of ribosomal pausing are temporary, ribosomes stalled on non-stop mRNAs have to be actively rescued and this process is considered essential for viability in bacteria^14^.

Trans-translation is a ubiquitous bacterial mechanism that resolves ribosome stalling caused by non-stop mRNAs^15^,^16^. It is operated by a nucleoprotein complex composed of SmpB (Small protein B)^17^,^18^ and tmRNA (transfer- and messenger-RNA), a highly structured RNA encoded by the *ssrA* gene^19^. Both components are highly conserved in bacteria and can be found in almost all sequenced bacterial genomes, except for a handful of eubacterial species and some insect endosymbionts^20^. The SmpB-tmRNA complex recognizes stalled ribosomes with a free A site into which the complex is loaded, thanks to the Alanine-tRNA-like structure of tmRNA, assisted by SmpB^21^,^22^,^23^. The unfinished polypeptide is then transferred onto tmRNA by transpeptidation, displacing tmRNA into the P site. The non-stop mRNA is released and degraded by RNase R, an exoribonuclease that interacts with the trans-translation complex^24^,^25^. Translation resumes using tmRNA as a new template. The messenger section of tmRNA bears a short open reading frame that encodes a degradation tag recognized by different proteases^26^,^27^,^28^, and also ends with a stop codon, allowing normal termination of translation. Trans-translation therefore not only resolves ribosome stalling, but also triggers the degradation of both the source of the problem (the faulty mRNA) and its consequences (aborted translation products, stalled ribosome subunits).

Trans-translation is highly mobilized in metabolically active bacteria, even in non-stressful conditions where at least one trans-translation event occurs per ribosome and per cell cycle in exponentially growing *Escherichia coli*^4^. Further highlighting the essential nature of ribosome rescue for the cell, alternate ribosome rescuing systems are widely found in the phylogeny and can compensate the loss of ribosome rescue by trans-translation^14^,^29^. Two have been identified so far: ArfA^30^ and ArfB^31^ (Alternative rescue factors A and B). Both are only able to recognize stalled ribosomes and promote their dissociation, while lacking mRNA and protein degradation signals. In a small number of bacterial species where none of those alternatives have been found, trans-translation is essential and neither *ssrA* nor *smpB* genes can be deleted^32^,^33^,^34^,^35^,^36^,^37^. Interestingly, all those species are pathogens, suggesting that the loss of those redundant, less efficient ribosome rescue factors makes trans-translation a target for antibiotics that could spare the normal microbiota.

*Legionella pneumophila* is another pathogen that infects the human alveolar macrophage. It is a gram-negative bacterium responsible for epidemic pneumonia outbreaks. It is ubiquitous in freshwater environments where it infects and multiplies within eukaryotic hosts such as amoebas^38^. *L. pneumophila* can also infect humans by accident when contaminated water is aerosolized by man-made equipment (such as cooling towers, spas, showers or humidifiers) and inhaled. *L. pneumophila* infects alveolar macrophages in the same way it infects amoebas^39^, leading to a severe pneumonia called Legionnaire’s disease. Trans-translation had not yet been studied in *L. pneumophila* but analysis of available sequenced genomes indicates that all *Legionella* species lack the alternative rescue factor ArfA, and that except for a few species rarely found in a clinical setting (*L. drancourtii, L. quateirensis, L. tunisiensis* and *L. feeleii*), the majority also lacks ArfB. Several attempts to disrupt the *ssrA* or *smpB* genes in *L. pneumophila* have failed, suggesting that trans-translation might be essential in that pathogen^40^,^41^. Thus, trans-translation appears as a prime target for this intracellular pathogen that target the human alveolar macrophage.

We here used an innovative approach to confirm this result and study the physiological consequences of the loss of trans-translation activity in *L. pneumophila*. We report the phenotypes associated with the loss of trans-translation in *L. pneumophila*, namely in terms of multiplication in rich medium, infection of amoebas and human macrophages, antibiotic sensitivity and cell morphology. Our results support the potential of targeting trans-translation for therapeutic use against *L. pneumophila*.

## Results

### Construction of an inducible allele of *ssrA* in *L. pneumophila*

**Figure 1:**
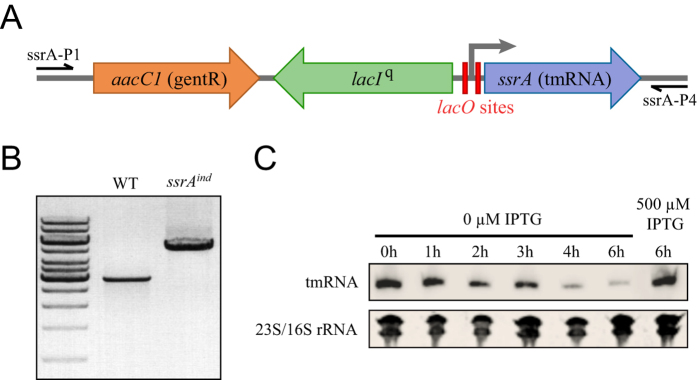
Contruction of *ssrA*^*ind*^, an *L. pneumophila* mutant in which *ssrA* is under repression by the LacI^q^ repressor. (**A**) Schematic diagram of the IPTG-inducible allele *ssrA*^*ind*^. A gentamicin resistance gene *aacC1*, the *lacIq* gene and a modified *ssrA* promoter with two LacO sites (red boxes) were inserted upstream of the predicted *ssrA* transcription start site. (**B**) PCR verification of the construction using primers ssrA-P1 and ssrA-P4. (**C**) Northern-blot analysis of tmRNA expression in the wild-type strain and the *ssrA*^*ind*^ mutant. Bacteria were cultivated with IPTG in solid medium then cultivated for the indicated duration in IPTG-free liquid medium or with 500 μM IPTG. Ethidium-bromide stained ribosomal RNA were used as loading controls.

Repeated attempts to generate a deletion mutant of the *ssrA* gene by allelic exchange with a kanamycin resistance marker failed to generate kanamycin-resistant transformants. We thus constructed a conditional mutant allele of *ssrA* by placing its expression under the control of the LacI^q^ repressor. We assembled a synthetic construct in which we inserted a first lac operator (*lacO*) repressor site between the putative −10 and −35 box of the *ssrA* promoter and a second *lacO* site between the −10 box and the *ssrA* transcription start site (Fig. 1A). Upstream of this modified promoter we also inserted a marker conferring gentamicin resistance for selection and a constitutively expressed *lacIq* gene. We introduced this synthetic construct in *L. pneumophila* by natural transformation and, in the presence of IPTG, we could isolate colonies in which the wild-type *ssrA* allele was effectively replaced by the inducible allele of *ssrA*, termed *ssrA*^*ind*^ (Fig. 1B). Despite the high stability of tmRNA, removal of IPTG leads to decreasing tmRNA levels over time (Fig. 1C) indicating that in the absence of IPTG, *ssrA*^*ind*^ expression is effectively under repression by LacI^q^.

### tmRNA is essential for axenic growth of *L. pneumophila*

**Figure 2:**
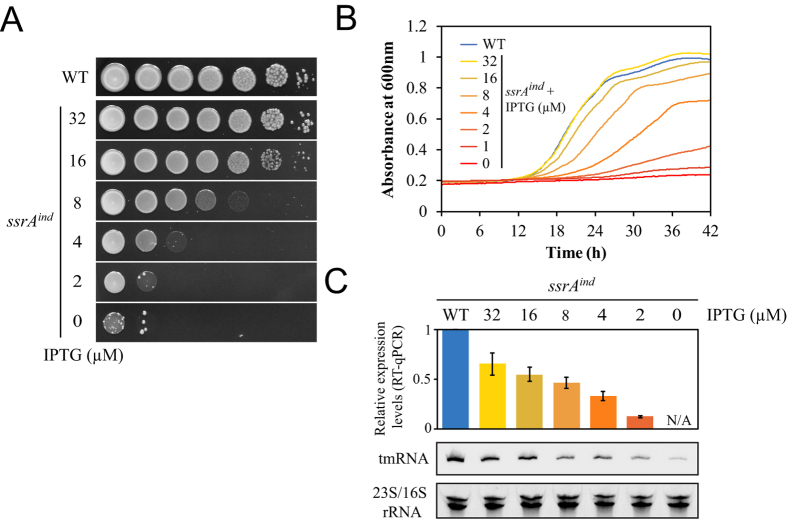
tmRNA is essential for growth of *L. pneumophila* in rich medium. (**A**) Serial, ten-fold dilutions of suspensions of the wild-type and the *ssrA*^*ind*^ mutant grown with IPTG were spotted on CYE solid medium containing various concentrations of IPTG. (**B**) Growth curve of the wild-type and the *ssrA*^*ind*^ mutant in AYE liquid medium containing various concentrations of IPTG. (**C**) Northern-blot and RT-qPCR analysis of the relative abundance of tmRNA after growth on solid medium. Ethidium-bromide stained ribosomal RNA were used as loading controls. RT-qPCR expression data are average (+/− standard deviation) from three quantifications.

Growth of the *ssrA*^*ind*^ mutant was assessed in the presence of varying concentrations of IPTG in standard *L. pneumophila* liquid medium AYE and on solid CYE medium. Ten-fold serial dilutions of an *ssrA*^*ind*^ mutant were spotted on CYE plates containing two-fold decreasing concentrations of IPTG (Fig. 2A). At concentrations of IPTG from 500 μM and down to 32 μM the *ssrA*^*ind*^ mutant could grow and produce colonies identical to those produced by the wild-type strain (Fig. 2B). Analysis of tmRNA expression by Northern-blot and quantification by real-time quantitative PCR (qPCR) revealed that at 32 μM expression of tmRNA is slightly lower than in the wild-type strain (expression of 0.65+/−0.11 relative to 1 in the wild-type strain). Increasing IPTG levels to 500 μM did not increase tmRNA levels (0.65+/−0.02), indicating that at 32 μM expression is already fully derepressed and that introduction of the *lacO* sites weakened the tmRNA promoter (Fig. 2C). At concentrations below 16 μM the *ssrA*^*ind*^ mutant could no longer produce colonies. In the absence of IPTG, no growth was observed and only revertants that escaped LacI repression were obtained at a frequency of ~1.10^−6^. Exactly as on solid media, concentrations of IPTG below 32 μM could not support normal growth in liquid medium (Fig. 2B). tmRNA is known to be expressed at high levels, sometimes even detectable by ethidium bromide staining of total RNA analysed on polyacrylamide gels. Indeed, RNAseq analysis of total RNA had found *ssrA* as the most expressed non-rRNA gene in *L. pneumophila*^42^. Interestingly, the qPCR data also revealed that reducing the amount of tmRNA only by a two-fold factor already has dramatic consequences on the growth and viability (Fig. 2C). Thus, high expression levels of tmRNA are required for normal growth in *L. pneumophila*. We conclude that trans-translation is essential in this human pathogen.

### tmRNA-depleted *L. pneumophila* can be rescued by other ribosome release systems

**Figure 3:**
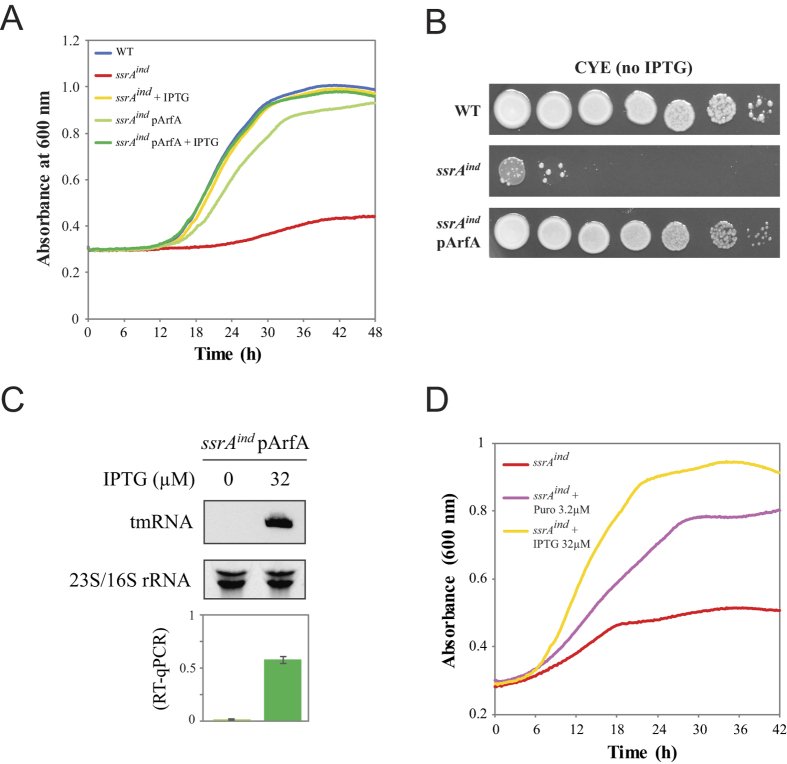
Ribosome release systems can compensate for the loss of tmRNA. Growth of the *ssrA*^*ind*^ mutant expressing the *E. coli arfA* gene in liquid AYE medium (**A**) and on solid CYE medium (**B**). (**C**) Northern-blot and RT-qPCR analysis of the relative abundance of tmRNA in the *ssrA*^*ind*^ mutant expressing the *E. coli arfA*. (**D**) Growth of the *ssrA*^*ind*^ mutant in the presence of subinhibitory concentrations of puromycin and in the absence of IPTG.

Trans-translation can release stalled ribosomes and also promotes the degradation of both the potentially hazardous faulty mRNAs and the aborted translation products. In order to determine the contribution of these activities to the normal of growth of *L. pneumophila* we determined whether other ribosome rescue systems could promote grow of the trans-translation deficient *ssrA*^*ind*^ mutant. The gene encoding the alternate ribosome rescue factor ArfA^30^ was cloned from *E. coli* into a plasmid to form pArfA and introduced in *L. pneumophila*. Ectopic expression of *E. coli arfA* had no effect on fitness, either in the wild type or in the *ssrA*^*ind*^ strain cultivated in presence of IPTG. In absence of IPTG, expression of ArfA could restore growth of the *ssrA*^*ind*^ mutant to a level close to that of the wild-type strain or the *ssrA*^*ind*^ mutant with 500 μM IPTG (Fig. 3A). Likewise, growth of the *ssrA*^*ind*^ mutant expressing ArfA was possible on solid media without IPTG but yielded smaller colonies than the wild-type strain or the *ssrA*^*ind*^ mutant with 500 μM IPTG (Fig. 3B). Northern-blot analysis and qPCR confirmed that tmRNA was not expressed in the *ssrA*^*ind*^/pArfA strain and that growth was not due to reversion of the repression (Fig. 3C). Therefore, ectopic expression of *E. coli arfA* can partially complement the loss of trans-translation in *L. pneumophila*. Puromycin is an antibiotic mimicking the structure of an aminoacyl-tRNA and induces spontaneous translation abortion, causing growth arrest at concentrations equal or higher than its MIC. At lower doses, the abortion of translation induced by puromycin can also rescue stalled ribosomes^43^, paradoxically allowing the growth of bacteria that have lost the functionality of their ribosome rescue mechanisms^30^. Indeed, growth of tmRNA-depleted *L. pneumophila* was possible in presence of a subinhibitory concentration of puromycin, albeit at a slower rate than the wild-type (Fig. 3D). Altogether the data indicate that the inability of the *ssrA*^*ind*^ mutant to grow in rich medium is primarily linked to loss of the ribosome rescue activity of trans-translation.

### Loss of tmRNA results in growth arrest and filamentation

**Figure 4:**
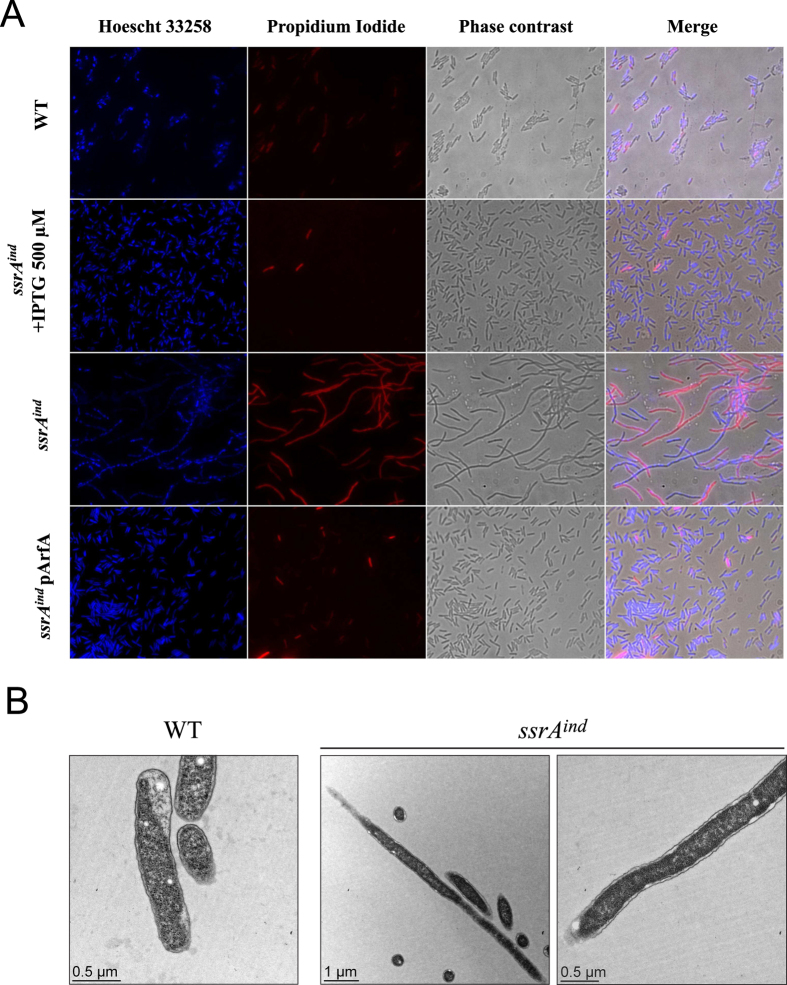
tmRNA-depleted *L. pneumophila* exhibit filamentation and impaired membrane integrity. (**A**) Fluorescence microscopy of the wild-type strain, *ssrA*^*ind*^ mutant and *ssrA*^*ind*^/pArfA grown for 24 hours (h) in AYE medium and stained with Hoescht 33258 and Propidium Iodide. (**B**) Electron micrographs of the wild-type strain and *ssrA*^*ind*^ mutant grown for 24 hours in AYE in the absence of IPTG.

In the absence of IPTG the *ssrA*^*ind*^ mutant could not produce colonies or grow normally in liquid medium, yet we observed a slight and variable increase in absorbance in liquid cultures suggesting that some growth could still occur in tmRNA-depleted cells (Figs 2B and 3A). Light microscopy revealed that under tmRNA depletion, the cells exhibited a striking filamentation phenotype (Fig. 4A). Absorbance increase in absence of IPTG is likely the result of that extensive cell elongation, which continued up to 48 h of culture, leading to filaments of a length up to several hundred micrometres long. Interestingly, DNA staining using the Hoescht dye was visible along the whole length of filaments, suggesting that DNA replication still occurs and results in multi-chromosomal cells. Propidium iodide (PI) staining was used to assess membrane integrity and is a widely used marker of cellular death^44^. A high proportion of tmRNA-depleted cells were stained by PI after 24 hours of culture, indicating that a large part of the filamentous tmRNA-depleted bacteria has a compromised membrane integrity and are probably dead. Interestingly, a significant number of filaments were still not stained by PI which suggests that their membrane is not compromised and that they could therefore still be alive even long after tmRNA depletion. This result was confirmed by regrowth assays in liquid medium where tmRNA-depleted cells could restart growth at a slower rate when IPTG was added 24 or 48 hours following removal of IPTG (Fig. S1A). The filamentous cells are thus likely able to resume division to some extent. Electron microscopy observations showed that no septa were visible in horizontal slices of filamentous cells (Fig. 4B) indicating that loss of trans-translation hampered establishment of the divisome. Ribosomes are visible as dark dots in electron micrographs and a higher ribosomal concentration was observed in the cytoplasm of the *ssrA*^*ind*^ mutant compared to the wild-type strain suggesting that tmRNA-depleted cells compensate the accumulation of stalled polysomes with synthesis of new ribosomes (Fig. 4B). This data indicates that the major consequence of loss of trans-translation is the inhibition of the cell division machinery. This may be due to a direct requirement of trans-translation to express components of the divisome at sufficient levels. *L. pneumophila* is also known to filament under nutrient scarcity in late stationary phase or under nutrient-limiting growth conditions^45^. The filamentation phenotype may thus also reflect the inability of the *ssrA*^*ind*^ mutant to use the nutrients provided by laboratory medium.

### tmRNA-depleted *L. pneumophila* are impaired for multiplication within eukaryotic hosts

**Figure 5:**
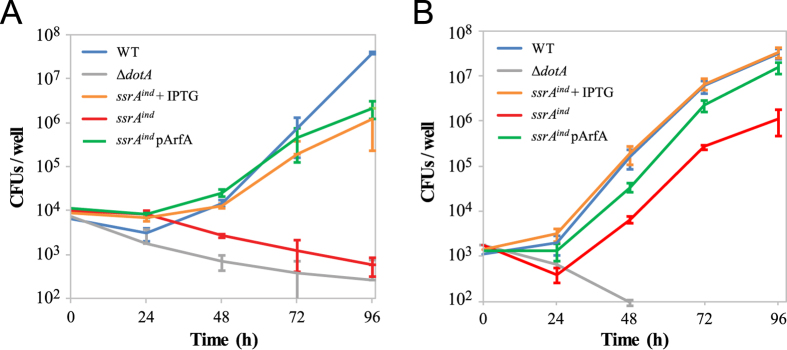
Growth of tmRNA-depleted *Legionella pneumophila* is impaired in eukaryotic hosts. Intracellular multiplication in *A. castellanii* (**A**) and U937 monocyte-derived human macrophages (**B**) was followed by plating samples on CYE plates containing IPTG and counting colony-forming units. Both charts represent the average of technical triplicates and are representative of three independent experiments. Error bars indicate standard deviation between the triplicates.

While trans-translation is evidently required for the growth of *L. pneumophila* in rich medium, it is unsufficient to conclude on its essentiality in the natural environment of the bacteria. The natural ecological niche of *L. pneumophila* is the intracellular replication vacuole that the bacterium establishes within its eukaryotic host, where it uses nutrients different than those found in the laboratory medium^46^. Evidence has shown that some stresses (heat, nutrient starving, sodium, chlorine) can hinder the culturability of the bacterium *in vitro*, but they nevertheless retain their ability to infect amoebas^47^,^48^,^49^,^50^. We thus tested the ability of the *ssrA*^*ind*^ mutant to infect, survive and replicate within amoebas and human macrophages. Wild-type, *ssrA*^*ind*^ and *ssrA*^*ind*^/pArfA strains were used to infect eukaryotic hosts at a low multiplicity of infection (MOI = 0.01 or 0.001), and sampling was performed daily to estimate the rate of bacterial multiplication. The avirulent *dotA* mutant, unable to replicate within cells was used as a negative control. In the amoeba *Acanthamoeba castellanii*, high-level expression of *ssrA*^*ind*^ allowed infection of the host and replication at a normal rate. In contrast, infection in absence of IPTG did not allow *L. pneumophila* to establish an infection cycle and led to a swift decline in bacterial charge, similar as in the *dotA* avirulent strain (Fig. 5A). Ectopic expression of *arfA* restored almost completely the virulence of the *ssrA*^*ind*^ strain in absence of IPTG (Fig. 5A). In monocyte-derived U937 human macrophages, high-level expression of *ssrA*^*ind*^ or *arfA* gave similar results, and in absence of IPTG, *ssrA*^*ind*^ was also impaired for multiplication within macrophages, albeit less affected than in amoeba *Acanthamoeba castellanii* (Fig. 5B). The *ssrA*^*ind*^ suffered a sharp decrease in bacterial count at 24 h, similar to the avirulent *dotA* mutant. Yet, following this the *ssrA*^*ind*^ mutant was able to grow but the infection yielded two orders of magnitude fewer cfu than that of the wild-type strain or of the *ssrA*^*ind*^ mutant in the presence of IPTG. Analysis of tmRNA expression by qRT-PCR showed that LacI^q^ repression was not very effective in this condition of infection, with tmRNA only three times less expressed in the absence of IPTG compared to with 500 μM of IPTG. This probably explains the significatively less strong phenotype of the *ssrA*^*ind*^ mutant compared to the infection of the amoeba host. Overall, the collected data show that trans-translation is not only required for growth in artificial rich medium but also for infection and replication within its natural and accidental hosts.

### Trans-translation promotes resistance to ribosome-targeting antibiotics

Trans-translation has recently been shown to mediate tolerance to a number of ribosome-targeting antibiotics^51^,^52^, although such data only exists for *E. coli* and not for any species in which the mechanism is essential. Thanks to our approach, we could also study the effect of a decreased expression of *ssrA* on antibiotic tolerance in *L. pneumophila*. The role of trans-translation was assessed by comparing the efficiency of different classes of antibiotics against the *ssrA*^*ind*^ mutant, in presence of excess IPTG (500 μM) or with an IPTG concentration (4 μM) that allows *L. pneumophila* to grow at 50% of its normal growth rate. Under such conditions, the levels of tmRNA are limiting and represent the closest possible situation to the absence of trans-translation. IC50, the concentrations at which half the growth was obtained compared to the condition without antibiotic, was used to estimate if there was a significant difference in antibiotic sensitivity when trans-translation activity was reduced. IC50 of ribosome-targeting antibiotics were significantly decreased (Mann-Wilcoxon U test, one-tailed, n = 6, p < 0.005) but not IC50 of Norfloxacin (p = 0.93), which targets the DNA gyrase involved in replication. The data are consistent with the situation is other species and confirmed that trans-translation is required to overcome the stress caused by translation-inhibiting antibiotics. Interestingly, when trans-translation activity is limiting, *L. pneumophila* also appears to be slightly more susceptible to rifampicin, an antibiotic that targets the RNA polymerase (Mann-Wilcoxon U test, one-tailed, n = 6, p < 0.01).

## Discussion

Despite being an efficient mechanism for ribosome rescue, trans-translation is not essential in model bacterial species. In *E. coli* or *Bacillus subtilis*, the deletion of *ssrA* or *smpB* is tolerated and compensated by alternative rescue factors like ArfA and/or ArfB^8^,^52^,^53^,^54^,^55^. In contrast, trans-translation was proven essential in a number of bacterial pathogens of high clinical importance that lack either one or both of the two known alternative rescue factors. (*Mycoplasma pneumoniae*^32^, *Mycoplasma genitalium*^32^, *Neisseria gonorrhoeae*^33^, *Haemophilus influenzae*^34^, *Helicobacter pylori*^35^, *Shigella flexneri*^36^, *M. tuberculosis*^37^). Our study was motivated by the fact that all sequenced *L. pneumophila* strains (and most *Legionella* spp.) were found to lack both known ribosome rescue factors alternative to trans-translation, suggesting that trans-translation might be essential and a potential therapeutic target. We developed here a conditional inactivation strategy with a chromosomal inducible allele replacement to verify this prediction. The constitutive *lacI*^*q*^ repressor is inserted upstream and in opposite orientation of the *ssrA* gene. The original promoter of the targeted *ssrA* gene is conserved but with *lacO* sites inserted between regulatory sequences (Fig. 1A). This leaves the gene itself untouched, allowing normal expression levels in absence of repression by LacI^q^. A more conventional approach is to delete the targeted gene while providing the same gene on a temperature-sensitive multicopy plasmid^56^. Our approach has the advantage of keeping the original genetic context of the targeted gene and its copy number. In the conditional *ssrA*^*ind*^ mutant, normal growth was obtained in presence of a soluble, readily available chemical inducer (IPTG), allowing us to grow cultures and then depleting them of tmRNA at will. This approach suffered from a few flaws, in particular the emergence of unrepressed mutants in cultures at a frequency of 1.10^−6^ (Fig. 2A), which required us to systematically check for revertants. Repression in the absence of IPTG was not complete, and probably caused by the extremely high basal transcription of the near-consensus promoter of *ssrA*, a fact confirmed by variability in repression strength depending on growth conditions, as seen during infection of human macrophages and discussed below. Yet, using this inducer-dependent expression system we show that high-level expression of tmRNA is essential for axenic growth of *L. pneumophila*, as evidenced by the inducer-dose-dependent growth in liquid or solid medium (Fig. 2). tmRNA, and by extension trans-translation, is therefore essential for growth of *L. pneumophila* in rich medium. Expression levels of tmRNA necessary to support normal growth appear slightly lower than wild-type levels (Fig. 2), suggesting that tmRNA is produced in excess in wild-type cells in normal growth conditions. This indicates that expression of tmRNA is high enough to not be limiting in case of exposure to less favourable conditions (e.g. starvation when exiting the host, exposure to harmful environmental settings or antibiotics).

Ectopic expression of the alternative ribosome rescue system ArfA, cloned from *E. coli*, restored the ability of tmRNA-depleted cells to grow normally and to infect eukaryotic hosts, albeit with a fitness cost. This proves that as in other species^33^,^36^, the release of the stalled ribosomal subunits is critical for survival and growth but not protein tagging and mRNA decay. We attempted to delete *ssrA* and *smpB* by allelic exchange in an ArfA-expressing strain but failed to obtain any mutants. In addition to the tmRNA level measurements that showed some residual amounts of transcript in depleted cells even in absence of IPTG, this result suggests that a low residual trans-translation activity probably is still present, and that *E. coli* ArfA by itself could not be sufficient to sustain *L. pneumophila* viability.

The inducer-dependent conditional expression system allowed us to analyse the consequences of loss of trans-translation. The striking, homogenous filamentation phenotype observed in tmRNA-depleted cells had never been reported and indicates that bacterial elongation can continue despite accumulation of stalled ribosomes, while cell division itself is quickly impaired (Fig. 4). Subpopulation of filamentous cells can be routinely observed in *L. pneumophila* cultures grown on solid medium^57^ or exposed to stressful conditions (antibiotics, UV light)^58^. In such cases the population exhibits widely varying lengths and the underlying mechanisms are poorly understood^59^. The filamentation induced by loss of trans-translation appears distinct as it is a more homogenous phenotype that increases overtime, with filaments several hundred micrometers long after 48 h of culture (Fig. S1B). A similar, systematic filamentous phenotype was previously observed when the activation of the stringent response was impaired in *L. pneumophila*. Indeed, deletion of *relA* and *spoT* (which both synthetise the stringent response alarmone ppGpp) induced filamentation in the post-exponential growth phase^60^,^61^. The stringent response is induced by RelA recognition of uncharged tRNAs loaded onto the A site of ribosome during starvation, and expression of *spoT* under inorganic phosphate starvation. Since tmRNA depletion should increase the number of stalled ribosomes with free A sites and slow global translation rates, it is possible that the pool of charged tRNAs stays high in tmRNA-depleted cells and that virtually no ribosome gets loaded with an uncharged tRNA. Meanwhile, the growth arrest may prevent phosphate levels from becoming limited in the medium. Both effects could lead to extremely low ppGpp levels and therefore mirror the filamentations phenotype of a *relA*/*spoT* mutant. An alternate hypothesis is that generalised ribosome stalling greatly reduces protein synthesis, preventing the production of the components of the cell division apparatus, whose levels have to be exactly regulated for normal division^62^. Additionally, levels of FtsZ, an essential component of the early division ring, are tightly regulated by direct recognition and degradation by the ClpXP protease^63^,^64^. In *E. coli*, reducing the level of expression of the *ftsZ* gene led to a very similar filamentation phenotype^56^. In *L. pneumophila*, deleting *clpP* also led to division issues^65^. ClpXP is one of the main operators for the degradation of SsrA-tagged unfinished polypeptides through the specific recognition of this tag by the adaptor SspB^28^. A sharp decrease in the number of SsrA-tagged substrates caused by tmRNA depletion could increase the availability of the protease and increase the degradation of FtsZ. We could not directly test this hypothesis, as specific antibodies against *L. pneumophila* FtsZ are not available. Translational fusions of FtsZ with an antibody-specific epitope tag (1X-, 2X- and 3X-Flag) induced an unwanted, systematic filamentation even in a wild-type background. Surprisingly, some of these filamentous bacteria were viable, able to form colonies and grow in liquid medium, showing that filamentation is not always associated with mortality in *L. pneumophila* and cannot explain alone the growth arrest observed during tmRNA depletion.

When placed in contact with a permissive amoeba host, the *ssrA*^*ind*^ mutant deprived of IPTG inducer failed to establish an infection cycle and the bacterial charge quickly dropped over time similarly to the avirulent *dotA* mutant. In monocyte-derived human macrophages, the phenotype was surprisingly milder, with a sharp increase in the usual, initial drop in bacterial charge (representing the subpopulation of bacteria from the inoculum who failed to establish a successful infection cycle) followed by successful intracellular multiplication. The multiplication was not due to the emergence of unrepressed revertants, as *ssrA*^*ind*^ bacteria sampled during intracellular multiplication remained unable to grow on solid medium without IPTG. We confirmed by RT-qPCR analysis that the growth observed was in fact due to a decreased repression of *ssrA* expression by LacI^q^ during infection of macrophages. The lower but non-null tmRNA levels explain the slower but successful infection of human macrophages by *ssrA*^*ind*^ in absence of IPTG, but the reason for the relaxed repression remains unexplained, as the RPMI infection medium supposedly does not contain any potential inducer (lactose). Despite this technical hindrance, infection profiles obtained here suggest that total or partial depletion of tmRNA and therefore inactivation of trans-translation indeed impairs the ability of *L. pneumophila* to multiply within its eukaryotic hosts.

No previous study had been conducted on the consequences of a reduction in trans-translation activity on stress sensitivity in species in which the mechanism is essential. Our system allowed to artificially reduce tmRNA levels, in order to assess its effect on sensitivity to replication, transcription and ribosome-targeting antibiotics. We induced a reduction in tmRNA levels that are limiting for growth and corresponding to a ~50% decrease in growth rate. We observed that for all tested ribosome-targeting antibiotics, but not for the gyrase-targeting norfloxacin, the decrease in trans-translation activity led to an increased antibiotic sensitivity. The strongest difference was observed with the macrolide erythromycin. Macrolides are the recommended treatment for Legionnaire’s disease in Europe and the US. A lower but significant difference was also observed with rifampicin, which targets the RNA polymerase and blocks transcription. Rifampicin activity probably increases the number of stalled ribosomes: first, by blocking unfinished transcription complexes, in which the stop codon has not been transcribed yet; and second, by reducing the availability of new transcripts for ribosomal subunits, which have an increasing chance over time to load onto an older, degraded, non-stop mRNA. Although there is a debate on the clinical efficiency of using a double antibiotic regimen for Legionnaire’s disease, some evidence of an increased efficiency has led to the frequent administration of rifampicin in association with a macrolide (erythromycin or azithromycin). Therefore, our results show that blocking trans-translation not only strongly impairs fitness and virulence of *L. pneumophila*, but it also increases the efficiency of two antibiotic drugs already used to treat Legionnaire’s disease.

In conclusion, trans-translation represents an interesting target for development of new antibiotic molecules targeting *L. pneumophila* and this species may be susceptible to recently discovered small molecule inhibitors of trans-translation^66^.

## Materials and Methods

### Chemical, growth conditions and culture media

*L. pneumophila* strains were grown either in AYE liquid media (ACES [N-(2-acetamido)-2-aminoethanesulfonic acid]-buffered yeast extract) or CYE solid media (ACES-buffered charcoal yeast extract). When necessary, IPTG (isopropyl β-D-1-thiogalactopyranoside) was used at concentrations ranging from 500 μM to 2 μM in either medium. When appropriate, gentamicin (7 μg/mL) or chloramphenicol (5 μg/mL) were added to the medium. Strains were stored in AYE supplemented with 15% glycerol at −80 °C, and were cultivated weekly by incubation on solid plates for three days at 37 °C. Fresh cultures used for all experiments were obtained by patching an inoculum on a new plate and incubating it for 24 h. tmRNA depletion was induced by resuspending fresh patches from a solid culture into liquid medium without IPTG. Growth in liquid medium was performed in 13 mL capped tubes containing 2–3 mL of medium at 37 °C with shaking (200 rpm). Alternatively, growth in 96-well plates, 100 μL/well, was monitored by measuring absorbance in a Tecan Infinite M200Pro temperature-controlled plate reader. Every ten minutes, the plate was agitated for 60 s with orbital shaking and 60 s with lateral shaking (5 mm amplitude each) and absorbance was measured at 600 nm. *E. coli* was grown on GL medium (Lysogeny broth with agar). Chloramphenicol (25 μg/mL), ampicilline (100 μg/mL) and X-gal (20 μg/mL) were added when needed.

### Strains and plasmids construction

All strains used in this study are derived from the *L. pneumophila* Paris clinical isolate CIP107629. Oligonucleotides used are listed in Supplementary Table 1, and plasmid sequences are available upon request. The conditional *ssrA*^*ind*^ mutant was constructed as follows. First, a 2 kb-long 5′ region flanking *ssrA* was amplified by PCR (Phusion DNA Polymerase, Thermo Scientific) using primers complementary to a gentamicin resistance cassette. Overlapping PCR was used to assemble this fragment with a PCR product containing an *aacC1* gentamicin-resistance cassette obtained from pXDC18. Second, a 2 kb-long region encoding *ssrA* (without its promoter) and its 3′ flanking region was amplified using primers containing a sequence complementary to *lacI*^*q*^ and a modified *ssrA* promoter with two high-binding lacO sites between the putative −35 and −10 transcription sites, and between the −10 and the +1 transcription sites. That modified promoter associated with *ssrA* form the *ssrA*^*ind*^ allele. Overlapping PCR was used to assemble a DNA fragment consisting of the *lacI*^*q*^ gene and the *ssrA*^*ind*^ + 5′ region product. Each assembled PCR products were cloned into pGEM-T Easy (Promega). The two resulting plasmids were then digested with AatII and XmnI restriction enzymes (New England Biolabs) and products of interest were separated by agarose gel electrophoresis and purified using a Qiaquick gel extraction kit. A 2562 nt fragment (XmnI-3′region-*gentR*-AatII) and a 5083 nt fragment (AatII-*lacI*^*q*^*-ssrA*^*ind*^-3′region-pGEM-T Easy backbone-XmnI) were ligated using T4 DNA ligase (NEB), transformed into *E. coli*. The pGEM-T-*ssrA*^*ind*^ plasmid obtained was used to transform a hypercompetent *L. pneumophila* Paris *comR* mutant^67^ on a CYE plate containing 500 μM IPTG. Gentamicin-resistant *L. pneumophila* Paris *ssrA*^*ind*^ *comR* transformants were obtained and clones were reisolated on a CYE plate containing gentamicin and 500 μM IPTG. Genomic DNA was extracted using the Wizard Genomic DNA purification kit (Promega) and used to transform a wild-type *L. pneumophila* Paris strain as previously described. *L. pneumophila* Paris *ssrA*^*ind*^ mutants were obtained by natural transformation and purified on a CYE plate containing 500 μM IPTG.

The pArfA plasmid was constucted as follows. The alternative ribosome rescue factor gene *arfA* was cloned from *E. coli* K-12 (strain DH5-α λpir) into pX5, a pMMB207C derivative, under the control of a constitutive promoter, to form the plasmid pArfA. pArfA was introduced by electroporation into *L. pneumophila* str. Paris WT and *ssrA*^*ind*^ and chloramphenicol-resistant transformants were selected.

### RNA extraction and quantification

Bacteria were grown for 3 days on CYE containing different concentrations of IPTG. Alternatively, bacteria were obtained by sampling supernatants from macrophage infections (performed as described below). At various time points, cells were centrifuged and resuspended in 100 μL TEL (Tris 10 mM, EDTA 1 mM, Lysozyme from chicken eggs 10 mg/mL, pH 7) on ice. Cell suspension was mixed with 1 mL commercial tri-reagent solution (acid guanidinium thiocyanate-phenol-chloroform) and lysed by repeated pipetting. Lysis was pursued by 15 min of incubation at 65 °C, then the samples were centrifuged (3′ at 5000 g). RNA extraction was continued by mixing the supernatant with an equal volume of chloroform and centrifuging (3′ at 5000 g). The supernatant was collected and precipited by isopropyl alcohol. The pellets were washed by 80% ethanol, dried, then resuspended in RNase-free water. RNA sample purity and concentration were determined by spectrophotometric analysis on a NanoDrop 2000 UV-Vis Spectrophotometer (Thermo Scientific).

### Northern-blot analysis

Northern-blot analysis was performed as previously described^42^. One microgram of total RNA in TBE (Tris, Borate, EDTA)-Urea denaturing buffer (Thermo Fischer) was loaded per lane and run on denaturing urea 6% acrylamide gel in TBE buffer. Total RNAs were visualized by ethidium bromide staining to check for equal loading of the lanes. RNAs were transferred to a charged nylon membrane (Amersham Hybond-N+, GE Healthcare Science) by electrophoretic transfer (30 min, 300 mA) and then cross-linked to the membrane by UV irradiation. Membrane were hybridised at 42 °C with 5′-biotinylated oligonucleotide probes (5 nM) for tmRNA in ULTRAhyb Ultrasensitive Hybridization Buffer (Ambion). Following overnight hybridisation, the membranes were washed twice in 2X SSC buffer containing 0.1% SDS at 65 °C according to the ULTRAhyb manufacturer instructions. Probed membranes were revealed using horseradish peroxidase-conjugated streptavidin and enhanced luminol substrate (Chemiluminescent Nucleic Acid Detection Module, Pierce). Luminescence signals were acquired using an imaging workstation equipped with a charge-coupled device camera (Thermo Scientific).

### Real-time quantitative PCR analysis

RNA were extracted as described above and traces of genomic DNA were removed by a DNase I treatment. RNA were purified on silica-based column (Zymo Research). RNA (2 μg) were reverse transcribed with RevertAid H Minus First Strand cDNA Synthesis Kit (Thermo Scientific) and used as template for real-time PCR with Premix Ex Taq™ (Tli* RNase H Plus) (TaKaRa) on LC480 instruments (Roche). Data were analyzed with the ΔCt method using 16 S rRNA as a reference. Amounts of tmRNA were expressed relatively to the wild-type strain.

### Infection of eukaryotic hosts

*A. castellanii* cells were routinely cultivated in PYG medium (Peptone-Yeast Extract-Glucose) at 30 °C. Four hours before infection, cells were incubated for 10 min on ice to allow detachment from the flask, then collected, centrifuged, and resuspended in A.c. buffer^68^. *A. castellanii* cells were plated in 24-well plates at a concentration of 10^6^ cells/well (three wells per tested strain and per condition). *L. pneumophila* strains were resuspended in A.c. buffer with or without IPTG (starting tmRNA depletion at that point) 4 hours before infection and incubated at 30 °C. At T0 of infection, bacteria were added at a concentration of 10^4^ cells/well (multiplicity of infection 0.01), 500 μM IPTG was added to designated wells, and infection was started by centrifuging the plates for 10 min at 1000 rpm. Bacterial concentration of the inoculum, as well as possible presence of revertants was verified by plating 10-fold serial dilutions on both CYE and CYE containing 500 μM IPTG. Similarly, at each time point, an aliquot of the supernatant was sampled and 10-fold serial dilutions were plated on CYE and CYE containing 500 μM IPTG to measure bacterial concentrations.

U937 human monocytes were cultivated routinely in RPMI-1640 Glutamax medium (Thermo Fischer) at 37 °C, 5% CO2. Three days before infection, cells were resuspended in fresh medium with 100 ng/mL PMA (Phorbol 12-myristate 13-acetate, Sigma-Aldrich), in order to trigger their differentiation into macrophages, and plated into 24-well plates (one well per tested strain and per time point, with technical triplicates). Four hours before infection, PMA was washed off by replacing the medium thrice, and *L. pneumophila* strains were resuspended in RPMI with or without 500 μM IPTG (starting tmRNA depletion at that point) and incubated at 37 °C. Bacteria were added to the wells at a MOI of 0.001, 500 μM IPTG was added when indicated, and infection was started by centrifuging the plates at 1000 rpm for 10 min. Inoculum concentration was verified by spotting 10-fold dilutions onto CYE and CYE containing 500 μM IPTG. Subsequently, at each time point and for each condition, the whole supernatant of a well was collected into a 1.5 mL tube, and the cells were incubated into 200 μL sterile ultrapure water + Triton X-100 0,1% for 5 minutes. The cells were detached by pipetting and promptly combined to the supernatants, and centrifuged 5 min at 6000 g. The supernatant was removed and replaced by 100 μL sterile ultrapure water, and 10-fold dilutions of that suspension of bacteria and lysed cells were spotted onto CYE and CYE containing 500 μM IPTG. This method allowed to determine bacterial multiplication while accounting for intracellular bacteria, with a very low detection limit of 20 bacteria/well, but was not applicable to amoebae which proved more resistant to lysis.

### Light and epifluorescence microscopy

Bacteria were cultivated in AYE with or without 500 μM IPTG and sampled at 0, 24 and 48 h. As a control for cell death, wild-type cells were heat-killed (3 min at 70 °C). Live bacteria were resuspended in room-temperature phosphate-buffer saline and stained for 10 min with Hoescht 33258 (500 μg/mL) to stain DNA in all cells and Propidium Iodide (20 μg/mL) to stain RNA and DNA only in dead cells or living cells with impaired membrane integrity. Bacteria were then washed twice with PBS and spotted onto PBS-agar 2% pads on glass slides, covered by a coverslip and immediately observed with a Zeiss Axioplan 2 epifluorescence microscope. All pictures were taken with the same exposition (10 ms for phase contrast and 500 ms for both fluorescent stains) and large fields with numerous bacteria are presented to accurately display the phenotype of the population observed in each condition. Pictures were coloured, contrast-adjusted (except for the Propidium Iodide images) and assembled using the ImageJ software.

### Electron microscopy

Bacteria were cultivated in AYE with or without 500 μM IPTG and sampled at T = 24 h. Fixation was performed in 2.5% glutaraldehyde, 2% formaldehyde in 100 mM cacodylate buffer at pH7, followed by staining with osmium tetroxide, dehydration and embedding in epoxy resin. Slices were obtained with a microtome and observed on a Philips CM120 electron microscope. Pictures were contrast adjusted and assembled using the ImageJ software.

### Antibiotic sensitivity assay

*ssrA*^*ind*^ bacteria were cultivated in AYE in a Tecan Infinite M200pro plate reader as described above, in presence of two-fold increasing concentrations of antibiotics centred around the minimum inhibitory concentration, and in presence of either 4 or 500 μM IPTG. Every 10 min, absorbance was measured. The highest absorbance measurement obtained for each condition was used as reference to trace a sigmoid curve corresponding to maximum observed absorbance in function of antibiotic concentration. For each condition, we verified that the value of the data point retained for further analysis was realistic in view of the rest of the values in the growth curve, and that it was not an isolated outlier value. The mathematical function corresponding to the experimental values was obtained by non-linear regression using the QtiPlot software. IC50, the concentration at which 50% growth inhibition would be observed compared to the condition without antibiotic, was determined from the obtained mathematical function. The function used was y = Bottom + (Top-Bottom)/(1 + 10 ^ ((IC50-x)*HillSlope)), wherein “bottom” and “top” are fixed and defined as the lowest (highest concentration of antibiotic) and highest (no antibiotic) measured absorbance values; values for HillSlope (steepness of the curve) and IC50 were determined through a Levenberg-Marquardt normalized algorithm. This method led to values of R^2^ > 0.99 in all tested conditions and allowed to compare antibiotic sensitivity in both high- and low-levels of tmRNA conditions, despite different basal growth ability.

## Additional Information

**How to cite this article**: Brunel, R. and Charpentier, X. Trans-translation is essential in the human pathogen *Legionella pneumophila. Sci. Rep.* **6**, 37935; doi: 10.1038/srep37935 (2016).

**Publisher's note:** Springer Nature remains neutral with regard to jurisdictional claims in published maps and institutional affiliations.

## Supplementary Material

Supplementary Material

## Figures and Tables

**Figure 6:**
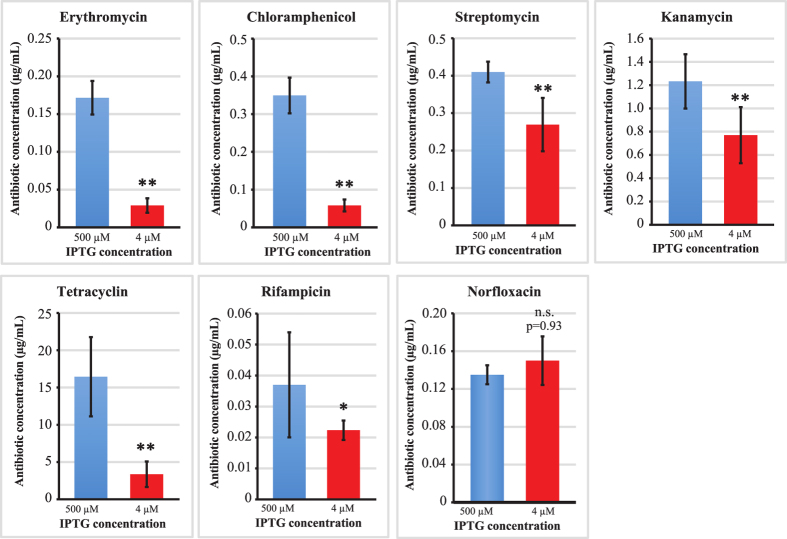
Antibiotic sensitivity of *L. pneumophila* with limited *ssrA* expression. The *ssrA*^*ind*^ strain was grown in AYE with either 500 or 4 μM IPTG and antibiotic sensitivity was assayed as described in Materials and Methods. The chart represents the average IC50 obtained in six independent experiments, and error bars indicate standard deviation between experiments. (**) = p < 0.005, (*) = p < 0.01, (n.s.) = non significative, Mann-Wilcoxon U test, one-tailed.
