# Supplementary material for: Trans-translation is essential in the human pathogen Legionella pneumophila
Source: Sci Rep. 2016 Nov 28;6:37935. doi: 10.1038/srep37935 (PMC5124942; doi:10.1038/srep37935)
Supplement: Supplementary Material [file srep37935-s1.pdf]

Supplementary material

Trans-translation is essential in the human pathogen *Legionella pneumophila*

Short title: trans-translation in *Legionella pneumophila*

Romain Brunel[1,2] and Xavier Charpentier[1,2*]

Supplementary Table 1. Oligonucleotides used in this study.

**Oligonucleotide name**	**Sequence**	**Comments**
ssrA-P1	GATAGCCTCGCCGATGAACTG	
ssrA-P2	TTCCACGGTGTGCGTCCATGGG<br>CAACATAAAATGCCGGCGTGTAAGC	Underlined:<br>gentR cassette adaptor
ssrA-P3-3	**TTGCTT**ATTGTGAGCGGATAACAAT<br>**TATAAT**TGTGAGCGGATAACAATTG<br>GGGGGCGACCTGGCTTCG	Underlined: lacO sites.<br>Bold: -35/-10 promoter sequences.
ssrA-P4	GTTCTGACTCTGAGGGCAGTAATTCC	
gnt-F	TTGCCCATGGACGCACACCGTG	
gnt-R	CTCCCCGCGCGTTGGCCGATTCATTA<br>AGTGCCACCTGGCGGCGTTG	Underlined: lacI^q adaptor (unused).
lacI^q-F	TTAATGAATCGGCCAACGCGC	
lacI^q-R	ATTATAATTGTTATCCGCTCACAATAA<br>GCAAAGAACCGTTATGATGTCGGCGC	Underlined: *ssrA*^{ind} promoter adaptor
tmRNA-NB	CCCTGAGTTTCGCGCCATAACCCGGGATA<br>GATTAGGAC	Northern-blot probe
lpptmRNA-RT-F1	CCGCTTATCGGTATCGAATC	Forward qPCR primer
lpptmRNA-RT-R1	CGCAAGTCCTCTGCCTTTAG	Reverse qPCR primer
16s-RT-F1	TGATGGTGGGGACTCTAAGG	Forward qPCR primer
16s-RT-R1	TTCATGGAGTCGAGTTGCAG	Reverse qPCR primer

A

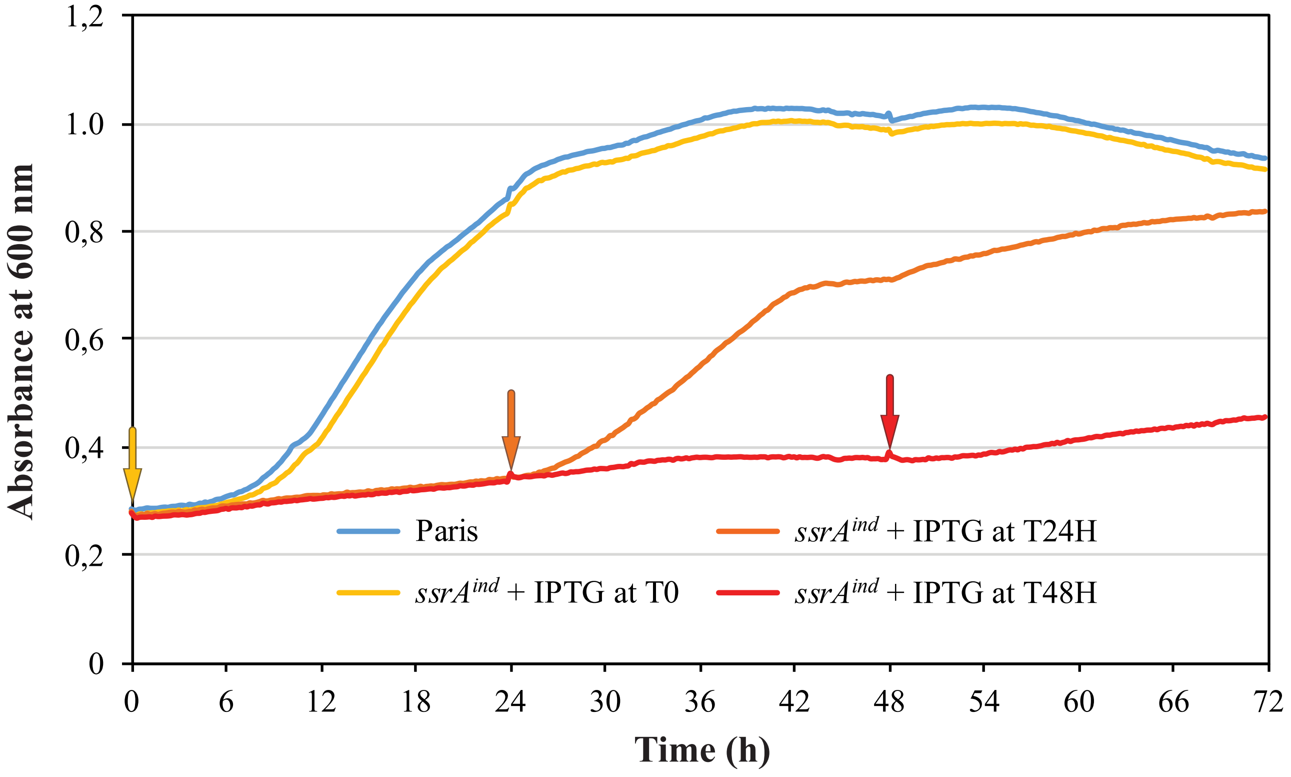

B

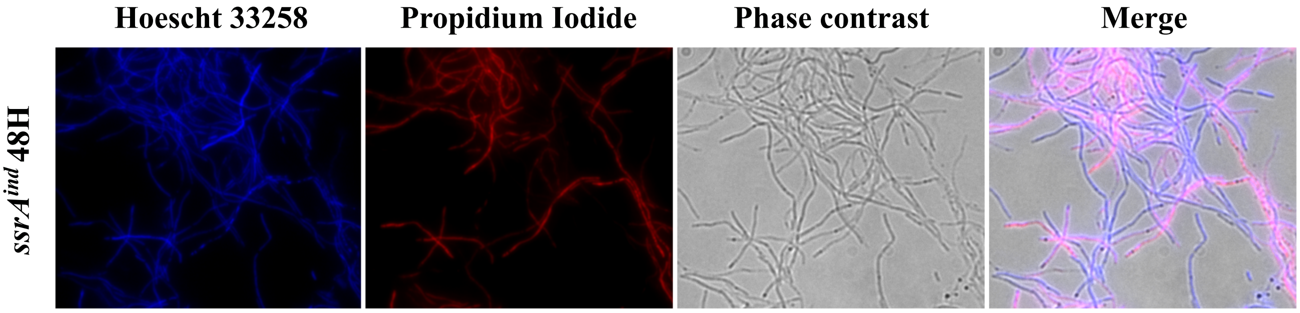

Figure S1. (A) Growth kinetics of the *ssrA*^{ind} mutant when IPTG is added at T=0h, 24h or 48h of culture (indicated by arrows). Regrowth is possible but the ability to resume growth decreases over time. (B) After 48h of culture in tmRNA-depleting conditions, the filamentation phenotype is even more marked than at 24h.
